# Local imperceptible adversarial attacks against human pose estimation networks

**DOI:** 10.1186/s42492-023-00148-1

**Published:** 2023-11-21

**Authors:** Fuchang Liu, Shen Zhang, Hao Wang, Caiping Yan, Yongwei Miao

**Affiliations:** School of Information Science and Technology, Hangzhou Normal University, Hangzhou, 311121 Zhejiang China

**Keywords:** Adversarial attack, Human pose estimation, White-box attack, Imperceptibility, Local perturbation

## Abstract

Deep neural networks are vulnerable to attacks from adversarial inputs. Corresponding attack research on human pose estimation (HPE), particularly for body joint detection, has been largely unexplored. Transferring classification-based attack methods to body joint regression tasks is not straightforward. Another issue is that the attack effectiveness and imperceptibility contradict each other. To solve these issues, we propose local imperceptible attacks on HPE networks. In particular, we reformulate imperceptible attacks on body joint regression into a constrained maximum allowable attack. Furthermore, we approximate the solution using iterative gradient-based strength refinement and greedy-based pixel selection. Our method crafts effective perceptual adversarial attacks that consider both human perception and attack effectiveness. We conducted a series of imperceptible attacks against state-of-the-art HPE methods, including HigherHRNet, DEKR, and ViTPose. The experimental results demonstrate that the proposed method achieves excellent imperceptibility while maintaining attack effectiveness by significantly reducing the number of perturbed pixels. Approximately 4% of the pixels can achieve sufficient attacks on HPE.

## Introduction

Although great success has been achieved using deep learning systems in various tasks, recent research has shown that neural networks are susceptible to small imperceptible perturbations called adversarial examples. Owing to the existence of adversarial examples, attacking a classifier becomes a search problem within a small perturbation around a target image. The reliability of neural networks is attracting increasing attention. Exploring adversarial attacks would benefit the understanding of deep learning models and the development of a more robust model. Although adversarial perturbations can effectively attack image classification networks, leading to incorrect predictions, relevant research on human pose estimation (HPE) has not been conducted.

Two issues remain to be solved regarding adversarial attacks on HPE networks. First, adversarial examples generated by perturbing an entire image are insufficiently imperceptible, particularly when the human-body region is easily detected by the human vision system. The tradeoff between the optimality and imperceptibility of adversarial attacks has already been implemented using minimum-norm attacks such as Carlini & Wagner attack (C&W) [1]. The goal of a minimum-norm attack is to minimize the perturbation strength while ensuring its success. An alternative to the minimum-norm attack is the maximum allowable attack that constrains the strength of the attack under an upper bound, such as the fast gradient sign method (FGSM) [2]. Both methods require the perturbation to be as small as possible. However, these two attacks focused on the entire image and depended on predefined iterations and the upper bound of the attack strength. Another study [3] generated a one-pixel adversarial perturbation based on differential evolution and successfully fooled a neural network by changing only a single pixel of the image to a specific value. Recent researches have focused more attention on local perturbations on images to overcome attack optimality and imperceptibility. Studies have shown that perturbing pixels limited to a small area can craft invisible attacks that are difficult to detect by the human eye. Second, HPE networks are a blend of classification and regression architectures, resulting in a difference between the objective function of adversarial attacks on HPE and its counterpart in image classification tasks. Methods based on boundary attacks and softmax cross-entropy cannot be applied to HPE.

To solve the two problems, we propose a novel method for crafting perturbations to critical pixels rather than a full image with appealing imperceptibility. Therefore, our method is an evolving version of the FGSM that considers attack optimality and imperceptibility. We reformulate a local invisible adversarial attack into an *l*_0_ optimization problem and provide a greedy algorithm for its optimization. The novelty of our study lies in both the research problem and the proposed solution. In particular, local invisible adversarial attacks on HPE have not been explored. We are also the first to propose a formulation that (1) considers both the perturbation strength and pixel selection of adversarial samples and (2) generalizes minimal local perturbation to HPE networks.

The proposed attack method aims to perturb human-body keypoints using a small number of perturbed pixels. In particular, we convert our problem into a maximum allowable attack under an *l*_0_ norm constraint in the keypoint regression framework and solve it using a greedy algorithm to find which pixels to perturb and what strength to add effectively and efficiently. Our optimization method can generate adversarial examples with high imperceptibility and maximum attack effectiveness.

The main contributions of this paper are as follows:

1. We studied imperceptible attacks on HPE and reformulated them into a problem of the maximum allowable attack under an *l*_0_ norm constraint in a regression form.
2. A greedy algorithm is proposed to solve the aforementioned *l*_0_-norm optimization by choosing pixels with less sensitivity to the human eye and maximizing the adversarial loss for keypoint regression.
3. Extensive experiments have shown that our method can successfully attack representative HPE networks with high efficacy and imperceptibility.

### Adversarial attack

According to the accessibility of the target models, adversarial attack methods can be divided into three types: white-box, gray-box, and black-box attacks. This study focuses on white-box attack methods that assume that adversaries can completely access the target models, including the model’s architecture, parameters, and gradients. On the contrary, black-box attacks have no access to the target model and can only observe its outputs. In contrast to the previous two attacks, gray-box attacks only assume access to the target model during the training phase or partial gradient information during the inference phase. In addition, an adversarial attack can be targeted, where the adversary’s goal is specified as a particular class *t*, or untargeted, where the adversary’s goal is any class other than the correct class.

FGSM [2] is the most representative work in white-box attacks, initially proposed by Goodfellow, and generates adversarial examples under the *l*_∞_-norm constraint to close to clean samples. The FGSM utilizes gradient information to update the adversarial example in one step along the direction of the maximum classification loss. Basic iterative method (BIM) [4] extends FGSM with an iterative scheme to craft adversarial perturbations through multistep updates. Projected gradient descent (PGD) [5] is similar to BIM, except that it randomly selects the starting point of an iterative attack. In essence, FGSM, BIM, and PGD belong to the category of maximum allowable attacks. DeepFool [6] generates the smallest perturbation while satisfying the target of a successful attack. DeepFool is a type of minimum-norm attack using the *l*_2_-norm. C&W [1] crafts an adversarial perturbation by optimizing regularization-based attacks. This method can generate adversarial examples under *l*_0_, *l*_2_, and *l*_∞_-norm constraints with minimal perturbation amounts. BIM, PGD, and C&W are commonly used white-box attack methods that work well for various datasets and domains.

Many studies have been conducted on black-box attacks, such as score-based [7], decision-based [8], and transfer-based [9] attacks. Although these methods have gradually improved the efficiency of transfer attacks or reduced the number of queries, black-box attacks still have a large performance gap between white- and black-box attacks.

### Imperceptibility

The true perceptual distance between two images, defined as how different a pair of images appears to humans, is nontrivial and cannot be easily computed or optimized. Fortunately, there exists many surrogate perceptual distances in the computer vision field, such as peak signal-to-noise ratio (PSNR), structural similarity (SSIM) [10], and learning perceptual image patch similarity (LPIPS) [11]. However, these metrics do not fully represent the perceptibility of the human eye. The traditional PSNR lacks structural representations and contradictions for human perception. SSIM focuses on the similarity between edges and textures to mimic human perception. However, a perception-driven distance function based on edges and textures is not ideal and performs poorly for nonstructural distortions. LPIPS [11] is more consistent with human perception than traditional methods. It was demonstrated that the LPIPS matched the human visual system well, without additional training weights. In this paper, we propose using all these as metrics to measure the perceptual similarity between two images. Further discussions and comprehensive studies can be found in the neural perceptual threat model [12].

### Attacks on HPE models

HPE can generally be categorized into regression- and heatmap-based methods. Regression-based methods such as DeepPose [13] and MaskRCNN [14] frame the HPE as a coordinate regression problem. However, regression-based methods are not widely used, because they lack spatial and contextual information. Heatmaps were first introduced in the joint training of a convolutional network and a graphical model for HPE [15], and rapidly became the most commonly used coordinate representation. Most state-of-the-art methods [16–18] attempt to improve network architectures for heatmap regression.

There are several evaluation metrics for HPE, such as object keypoint similarity (OKS) [19], percentage of correct keypoints [20], average precision (AP), and average recall (AR).

Little research has been conducted on adversarial perturbations in HPE, particularly adversarial attacks on human-body keypoint detection. A recent study [21] evaluated the robustness of most existing HPE models using various data corruptions such as blur and pixelation. Jain et al. [22] presented a study of adversarial attacks on HPE models and evaluated their robustness. Liu et al. [23] provided solutions for adversarial attacks and defenses against human activity recognition. In addition, these studies [24–26] achieved good attack effects on the adversarial perturbations of the human skeleton from the perspective of human action recognition. Although most of the aforementioned studies demonstrated that adversarial attacks are truly a threat to vision tasks based on HPE, their focus is not on imperceptible adversarial attacks on 2D HPE.

## Methods

In this section, we briefly introduce the optimization problem for imperceptible adversarial attacks on HPE and provide a greedy algorithm for optimization.

The idea behind this is that imperceptible adversarial attacks can be alternatively optimized using two suboptimal problems: strength refinement and pixel selection. In perturbation strength refinement, we use iterative gradient methods such as FGSM to optimize the attack strength. Subsequently, we added a perturbation with an optimized strength to select pixels for the target images. However, pixel selection optimization is *l*_0_ norm optimization, and hence, NP-hard in general. Therefore, we propose a greedy-based method to optimize which pixels to modify based on a sensitivity that is consistent with the human eye.

**Fig. 1:**
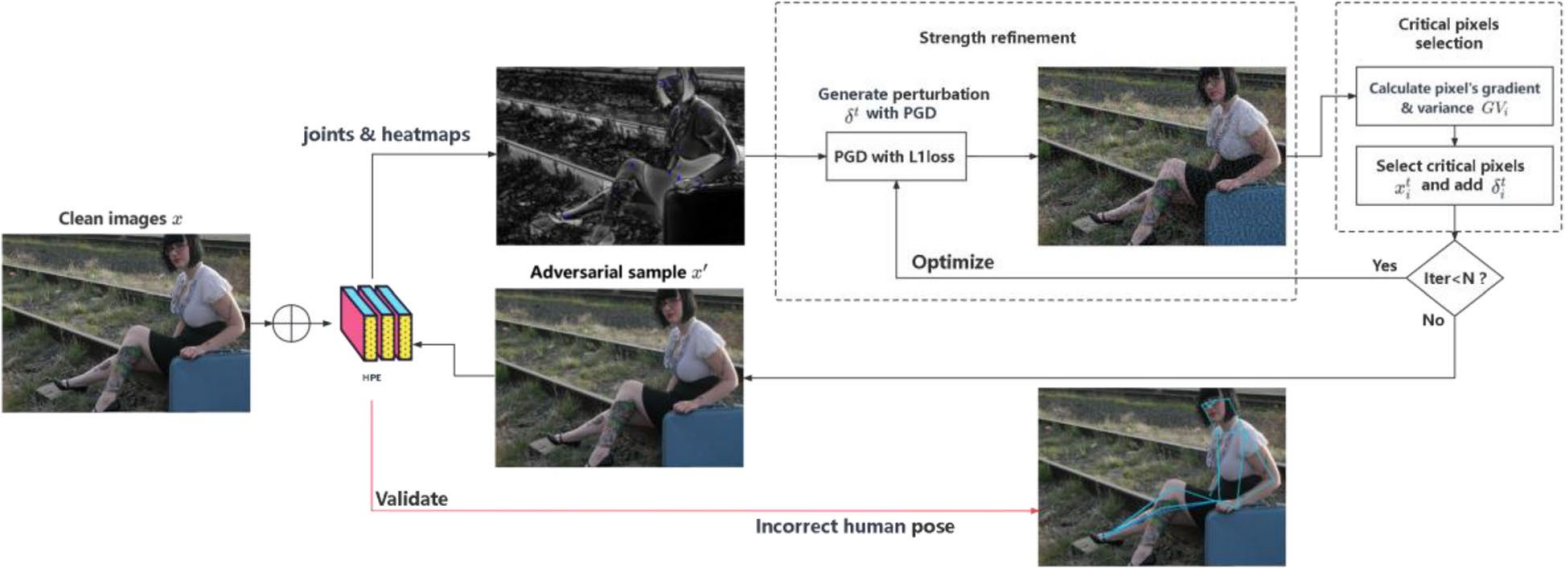
Overview of the proposed method. We frame local imperceptible adversarial attacks on HPE into two alternative optimizations. One is for perturbation strength refinement; the other is for critical pixel selection. Strength refinement is used for finding what strength to add, while pixel selection is used for finding which pixels to modify. The process is repeated until the iteration reaches the predefined value

Figure 1 illustrates the pipeline of the proposed local imperceptible adversarial attack on HPE. Given a clean image, a PGD attack is performed to compute the perturbation strength. Only the top *k* pixels based on our sensitivity metric (a weighted sum of gradients and variances) can be selected for the next iteration of strength refinement. Pixel selection and strength refinement were alternately performed to complement each other. The entire attack process is iterative and converges to a predefined upper bound of the perturbed pixels.

### Problem formulation

The problem of interest is as follows: Let *F*(*x*; *θ*) be a heatmap-based HPE model, where *x* ∈ *X* is the input image, and the output is keypoint heatmaps *H* = {*h*_1_*, h*_2_*..., h*_*n*_} for *n* human joint locations.

Given an image *x*, we aim to find a minimal set of pixels that can generate an adversarial image *x*^′^ to attack the network *F*(*x*; *θ*). We express the selection of pixels in *x* for perturbation by a binary indication vector **a** = [*a*_1_*, ..., a*_*N*_]^*T*^ ∈ {0*,*1}^*N*^ where *a*_*i*_ is 1 if the *i*^*th*^ pixel is selected, and 0 otherwise. *H*^*t*^ is the ground truth pose from the validation set. Suppose that *δ* = [*δ*_1_ *,... ,δ*_*N*_]^*T*^ ∈ **R**^*N*^ is the perturbation strength vector. In particular, **a**_0_ refers to the initial status of the pixel selection, and **x**_0_ refers to the clean image. The loss function for untargeted attacks can be expressed as

1 $$J\left(x,a,\delta \right)={\Vert F({x}_{0}+a\odot \delta ;\theta )-{H}^{t}\Vert }_{2}^{2}$$

The process of generating the minimal perturbation on HPE can be formulated as

$${min}_{a}{\Vert a\Vert }_{0}$$

2 $$s.t.\;{max}_{a,\delta\;}J\left(x,a,\delta\right)\;and\;{\Vert\;x-{x}_{0}\Vert\;}_{\infty\;}\le\;\eta$$

Where ${x}_{0}$ represents the original input to the HPE, which is the input data without any perturbation, $a$ represents the perturbation term, $\delta$ denotes a set of parameters that restrict or control the perturbation $a$ to ensure that the generated perturbation is minimal under certain constraints, $\eta$ is a constant representing the maximum *l*_0_ norm distance between the input data $x$ and the original input data ${x}_{0}$.

However, it is well known that *l*_0_ norm optimization is generally notorious and NP-hard. Thus, we convert Eq. (2) into a constrained optimization problem using the maximum allowable attack form as follows:

$${max}_{a,\delta }J\left(x,a,\delta \right)$$

3 $$s.t.\;{\Vert x-{x}_{0}\Vert }_{\infty }\le \eta , {\Vert a-{a}_{0}\Vert }_{0}\le \zeta$$

We apply a first-order approximation:

4 $$J\left(x,a,\delta\right)=J\left(x_0,+a\odot\delta,a_0+\Delta a,\delta_0+\Delta\delta\right)\approx J\left(x_0,a_0,\delta_0\right)+\nabla_xJ\left(x_0,a_0,\delta_0\right)\cdot\Delta x+\nabla_aJ\left(x_0,a_0,\delta_0\right)\cdot\Delta a=J\left(x_0,a_0,\delta_0\right)+\nabla_xJ\left(x_0,a_0,\delta_0\right)\cdot a\odot\delta+\nabla_aJ\left(x_0,a_0,\delta_0\right)\cdot\Delta a$$

Using a first-order approximation, we approximated the solution of Eq. (3) by decomposing it into two optimization problems (*strength refinement* and *pixel selection*), that described in Eqs. (5) and (7).

$${max}_{\Delta x}J\left(x_0,a_0,\delta_0\right)+\nabla_xJ\left(x_0,a_0,\delta_0\right)\cdot\Delta x$$

5 $$s.t.\;{\Vert \Delta x\Vert }_{\infty }\le \eta$$

Similar to FGSM, the solution of Eq. (5) is given by

6 $$x=x_0+a\odot\delta=x_0+\eta\cdot a\odot sign(\nabla_aJ\left(x_0,a_0,\delta_0\right))$$

The **a** can be optimized subsequently using Eq. (7).

$${max}_{\Delta a}J\left(x_0,a_0,\delta_0\right)+\nabla_aJ\left(x_0,a_0,\delta_0\right)\cdot\Delta a$$

7 $$s.t.\;{\Vert \Delta a\Vert }_{0}\le \zeta$$

For Eq. (7), it is *l*_0_ norm constraint optimization. To solve this problem, we applied a greedy algorithm for optimization. Thus, the optimization of Eq. (3) can be solved using Eqs. (5) and (7) alternately as follows.

### Sensitivity-based pixel selection

To solve Eq. (7), an efficient greedy algorithm is introduced to find pixels that modify and maintain imperceptibility. In particular, we ranked all pixels according to their sensitivities to the human eye and selected the less sensitive ones to be perturbed. The process is iterative and ceases when the number of selected pixels reaches *ζ*.

According to the observations of the contrast masking theory [27] in image processing, the human eye is usually more sensitive to pixel changes in low-variance areas than in high-variance areas [28]. Thus, we define a sensitivity that is consistent with that of the human eye using a weighted sum of gradients and variances, which is written as follows:

8 $${GV}_{i }= a\cdot g(|{\nabla }_{i}|) + b\cdot g({\sigma }_{i}^{2})$$

Where ∇_*i*_ represents the gradient of the adversarial loss function ∇_*x*_*J*(*x,* **a***, δ*) at pixel *i*, *σ*_*i*_^2^ represents the variance of the pixel with its *n* × *n* neighborhood. *a* and *b* are hyperparameters used to tradeoff attack effectiveness and imperceptibility, respectively. *g* is a function that scales the variables to the normalized range of [0, 1].

The philosophy of tuning *a* and *b* reflects the tradeoff between attack effectiveness and imperceptibility. When *b* is zero, the sensitivity focuses on attack effectiveness rather than invisibility. In contrast, increasing the value of *b* can reduce human perceptibility but reduces effectiveness. We give users the freedom to tweak the tradeoff of output samples between attack effectiveness and imperceptibility.

It is worth noting that even when *b* = 0, the adversarial samples generated by our method maintained good perceptual quality. This was because the selected number of pixels was sufficiently small. Taking an image resolution of 512 × 512 as an example, 10*k* pixels are finally selected, which means that less than 4% of all image pixels need to be perturbed. Additionally, the number of critical pixels was adapted to the resolution of the images. Pixel selection was implemented by ranking the sensitivity of the pixels and selecting the top *k* pixels.

### Greedy-based imperceptible adversarial attack via strength refinement and pixel selection

In this process, we select the top *k* pixels with a small sensitivity to perturbation and then add perturbations only to these pixels. We repeat the pixel selection and perturbation and output the final adversarial example.

For every iteration, we use the *l*_1_ norm regularized PGD method to attack the target human pose model, called *strength refinement*, which corresponds to Eq. (5). We solved this problem by using the iterative gradient method as the FGSM. Equation (1) can be rewritten as:

9 $$J\left(x,{x}^{\prime}\right)={L}_{H}\left(x.{x}^{\prime}\right)=\sum\nolimits_{i=1}^{n}{smooth}_{L1}({h}_{i}\left(x\right),{h}_{i}\left(x^{\prime}\right))$$

10 $$\delta=\epsilon\cdot sign(\nabla_xL_H)$$

where *h*_*i*_(*x*) is the predicted heatmap for the *i*^*th*^ joint of the clean image *x*. *h*_*i*_(*x*^′^) is the predicted heatmap for the *i*^*th*^ joint of the perturbed image *x*^′^. *η* is the perturbation constant. Then perturbation noise will be normalized into the range (−*ϵ, ϵ*), to further ensure the imperceptibility of the perturbation noise.

After strength refinement, we perform sensitivity-based pixel selection to refine the attack pixels as *pixel selection*, which corresponds to Eq. (7). Our proposed critical pixel-based perturbation method is different from the traditional PGD method, which perturbs all the pixels in the image. Instead, this method focuses on choosing the critical pixels in an image that are most vulnerable to attacks and are invisible to the human eye.

We note that some networks resize the input image to a fixed size during training and inference, which degrades the attack effectiveness. In our case, we added perturbations directly to the original images without changing the image size.

The steps of the greedy-based imperceptible attack via strength refinement and pixel selection are summarized in Algorithm 1.

**Figure:**
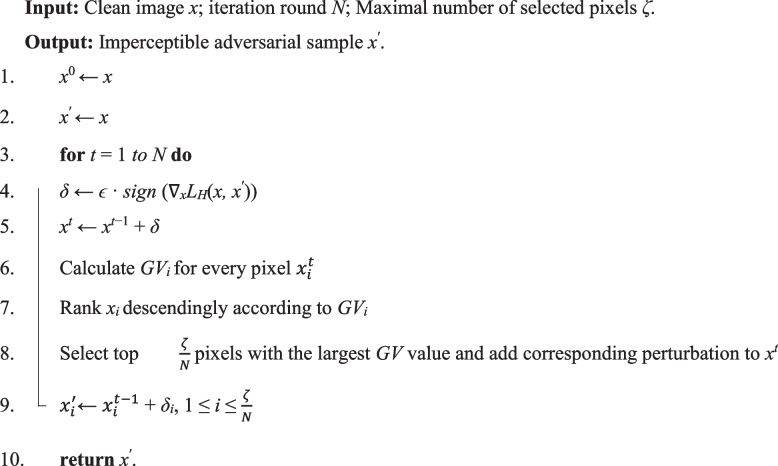
** Algorithm 1** Greedy-based local imperceptible adversarial attack

## Results and discussion

### Datasets and settings

#### Datasets

Extensive experiments were conducted using the MS COCO 2017 dataset [19]. The dataset contained 123287 images, of which 118287 were used for training and 5000 images were used for testing. The test set included 6352 human instances. All images in our experiments had 512 × 512 pixels.

#### Attack setup

All experiments were performed on an Intel CPU i7-11700 machine with 32 GB of RAM and a GeForce RTX 3060 GPU with 12 GB of memory under Ubuntu 20.04. We set the neighborhood size *n* = 5 and other hyperparameters as follows: BIM: *epoch* = 10, *eps* = 16, *ϵ* = *eps/*200, *ϵ*_*clip*_ = *eps/*255; PGD: *epoch* = 10, *eps* = 16, *ϵ* = *eps/*200, *ϵ*_*clip*_ = *eps/*255, *rand minmax* = *eps*; C&W: *c* = 1, *κ* = 0, *max iter* = 100, *learning rate* = 0*.*04; Ours: *epoch* = 10, *eps* = 160, *ϵ* = *eps/*200, *ϵ*_*clip*_ = *eps/*255.

#### Evaluation metric

The standard AP based on OKS, which is the same as that of COCO, was employed as the evaluation metric. The following metrics are reported: AP, AP^50^, AP^75^, AP^M^, AP^L^, AR, AR^50^, AR^75^, AR^M^, and AR^L^. We used the SSIM, PSNR, and LPIPS to evaluate the imperceptibility.

#### Baselines

We selected three state-of-the-art HPE methods as baselines: HigherHRNet [17], DEKR [18] and ViTPose [29]. For the model implementation, we directly used the release code and pretrained models from their official implementation websites.

### Quantitative comparison of perturbed accuracy and imperceptibility

Tables 1, 2, and 3 present the precision and recall of different architectures and attack types on the MS COCO dataset, respectively. From the results, we can conclude that both BIM and PGD attacks cause the AP to decrease significantly owing to their large perturbations. Consequently, they have worse imperceptibility (higher LPIPS, which means dissimilar) and degrade image quality with large noise (lower SSIM and PSNR, which means low quality; usually, SSIM is close to 1 and PSNR *>* 30 for high-quality images), as shown in Table 4. Our method was imperceptible and effective, reducing the precision of HigherHRNet to 60*.*5%, that of DEKR to 61*.*8%, and that of ViTPose to 62*.*0%. C&W has excellent imperceptibility but creates the weakest attacks, reducing the precision of HigherHRNet by 2%, DEKR by 1*.*6%, and ViTPose by 6*.*0%. In addition to weak attack effectiveness, C&W is slow for regularization-based unconstrained optimization. From Table 4, we observe that our method is very close to C&W in terms of the SSIM, PSNR, and LPIPS. ViTPose is a more powerful state-of-the-art model based on a transformer architecture. Unexpectedly, we found that earlier attack methods could effectively attack ViTPose. It is noteworthy that the values of SSIM, PSNR, and LPIPS on ViTPose are significantly different from the others because ViTPose has a different detection process from other networks by only detecting keypoints in cropped regions containing persons. Thus, we only added perturbation to the cropped person areas and computed the SSIM, PSNR, and LPIPS for these cropped images.

**Table 1:** Perturbed performance comparisons of different attack types on the COCO validation set for HigherHRNet

	AP	AP^50^	AP^75^	AP^M^	AP^L^	AR	AR^50^	AR^75^	AR^M^	AR^L^
Clean	0.671	0.862	0.730	0.615	0.761	0.718	0.885	0.768	0.651	0.814
BIM	0.499	0.706	0.538	0.411	0.627	0.543	0.723	0.579	0.441	0.684
PGD	0.463	0.723	0.494	0.395	0.568	0.541	0.770	0.573	0.454	0.661
C&W	0.651	0.842	0.710	0.578	0.760	0.695	0.865	0.742	0.610	0.815
Ours	0.605	0.802	0.658	0.516	0.739	0.653	0.826	0.696	0.552	0.796

AP^50^ is the AP at IOU = 0.5, AP^75^ is the AP at IOU = 0.75, AP^M^ is the AP for medium objects, AP^L^ is the AP for large objects

**Table 2:** Perturbed performance comparisons of different attack types on the COCO validation set for DEKR

	AP	AP^50^	AP^75^	AP^M^	AP^L^	AR	AR^50^	AR^75^	AR^M^	AR^L^
Clean	0.680	0.867	0.745	0.621	0.777	0.730	0.898	0.784	0.662	0.827
BIM	0.528	0.734	0.573	0.439	0.665	0.578	0.761	0.619	0.475	0.722
PGD	0.535	0.741	0.580	0.441	0.677	0.585	0.768	0.624	0.477	0.736
C&W	0.664	0.856	0.726	0.596	0.772	0.713	0.886	0.764	0.636	0.822
Ours	0.618	0.814	0.674	0.530	0.753	0.672	0.848	0.719	0.574	0.809

**Table 3:** Perturbed performance comparisons of different attack types on the COCO validation set for ViTPose

	AP	AP^50^	AP^75^	AP^M^	AP^L^	AR	AR^50^	AR^75^	AR^M^	AR^L^
Clean	0.693	0.835	0.757	0.631	0.788	0.732	0.860	0.787	0.661	0.834
BIM	0.444	0.613	0.475	0.357	0.566	0.512	0.665	0.545	0.405	0.661
PGD	0.445	0.613	0.473	0.355	0.571	0.515	0.669	0.546	0.406	0.665
C&W	0.633	0.795	0.690	0.578	0.716	0.684	0.827	0.734	0.616	0.782
Ours	0.620	0.800	0.675	0.542	0.734	0.670	0.824	0.718	0.582	0.794

**Table 4:** Imperceptibility of different attack types on HigherHRNet, DEKR and ViTPose

Metric Method Baseline	HigherHRNet			DEKR			ViTPose		
	SSIM↑	PSNR↑	LPIPS↓	SSIM↑	PSNR↑	LPIPS↓	SSIM↑	PSNR↑	LPIPS↓
BIM	0.559	23.993	0.469	0.575	24.137	0.478	0.883	16.810	0.137
PGD	0.559	23.993	0.469	0.574	24.198	0.468	0.882	16.792	0.137
C&W	0.934	31.148	0.090	0.934	31.165	0.089	0.919	17.928	0.107
Ours	0.928	31.299	0.118	0.924	31.293	0.130	0.935	22.775	0.095

### Ablation study

In this section, we study the effects of different loss functions, selected pixel numbers, and tradeoff hyperparameters. HigherHRNet was used as the baseline.

The results of the ablation study are presented in Tables 5, 6, 7, 8, 9 and 10. We can observe that (1) The adversarial loss function, based on the difference between the predicted and ground truth heat maps, provides the best attack results compared to other loss functions, such as the adversarial loss function. This result provides insight into crafting perturbations against keypoint regression networks; perturbing heatmaps is more effective than manipulating MSE-based regression losses. These three loss functions achieved very close imperceptibilities. (2) The increment of selected pixels (from 0*.*38% to 38% of 512^2^ pixels) gradually increases attack effectiveness but decreases imperceptibility, which also demonstrates the decent stability and flexibility of our local attack. (3) Parameters *a* and *b* provide a tradeoff between attack effectiveness and imperceptibility. *a* affects attack effectiveness, and *b* affects attack imperceptibility.

**Table 5:** Ablation study of attack effectiveness in terms of loss functions used in our method

	AP	AP^50^	AP^75^	AP^M^	AP^L^	AR	AR^50^	AR^75^	AR^M^	AR^L^
Clean	0.671	0.862	0.730	0.615	0.761	0.718	0.885	0.768	0.651	0.814
Ours (+ heatmap loss)	**0.605**	**0.802**	**0.658**	**0.516**	**0.739**	**0.653**	**0.826**	**0.696**	**0.552**	**0.796**
Ours (+ joints-MSE loss)	0.632	0.825	0.690	0.552	0.752	0.676	0.849	0.721	0.583	0.807
Ours (+ joints-OHKM-MSE loss)	0.623	0.823	0.679	0.539	0.748	0.669	0.846	0.713	0.572	0.804

**Table 6:** Ablation study of attack imperceptibility in terms of loss functions used in our method

	SSIM ↑	PSNR ↑	LPIPS ↓
Ours (+ heatmap loss)	**0.928**	**31.299**	**0.118**
Ours (+ joints-MSE loss)	0.949	31.388	0.096
Ours (+ joints-OHKM-MSE loss)	0.942	31.363	0.106

**Table 7:** Ablation study of attack effectiveness in terms of the number of selected pixels used in our method

	AP	AP^50^	AP^75^	AP^M^	AP^L^	AR	AR^50^	AR^75^	AR^M^	AR^L^
Clean	0.671	0.862	0.730	0.615	0.761	0.718	0.885	0.768	0.651	0.814
Ours (1000 pixels/0.38%)	0.645	0.834	0.701	0.569	0.759	0.690	0.860	0.737	0.603	0.812
Ours (5000 pixels/1.9%)	0.631	0.824	0.681	0.551	0.749	0.676	0.849	0.719	0.584	0.805
Ours (10000 pixels/3.8%)	0.605	0.802	0.658	0.516	0.739	0.653	0.826	0.696	0.552	0.796
Ours (20000 pixels/7.6%)	0.598	0.793	0.645	0.509	0.731	0.644	0.815	0.681	0.542	0.787
Ours (100000 pixels/38%)	**0.537**	**0.735**	**0.579**	**0.441**	**0.675**	**0.581**	**0.755**	**0.618**	**0.472**	**0.732**

**Table 8:** Ablation study of attack imperceptibility in terms of the number of selected pixels used in our method

	SSIM ↑	PSNR ↑	LPIPS ↓
Ours (1000 pixels/0.38%)	**0.950**	**32.245**	**0.093**
Ours (5000 pixels/1.9%)	0.946	31.343	0.108
Ours (10000 pixels/3.8%)	0.928	31.299	0.118
Ours (2000 pixels/7.6%)	0.893	30.508	0.170
Ours (100000 pixels/38%)	0.754	27.162	0.313

**Table 9:** Ablation study of attack effectiveness in terms of hyperparameters used in our method

	AP	AP^50^	AP^75^	AP^M^	AP^L^	AR	AR^50^	AR^75^	AR^M^	AR^L^
Clean	0.671	0.862	0.730	0.615	0.761	0.718	0.885	0.768	0.651	0.814
Ours (*a* = 1, *b* = 0)	**0.605**	**0.802**	**0.658**	**0.516**	**0.739**	**0.653**	**0.826**	**0.696**	**0.552**	**0.796**
Ours (*a* = 1, *b* = 0.5)	0.615	0.813	0.669	0.530	0.742	0.660	0.834	0.705	0.562	0.798
Ours (*a* = 1, *b* = 1)	0.639	0.833	0.698	0.562	0.761	0.692	0.866	0.741	0.606	0.814
Ours (*a* = 0.5, *b* = 1)	0.636	0.832	0.690	0.553	0.755	0.679	0.852	0.724	0.586	0.809
Ours (*a* = 0, *b* = 1)	0.642	0.834	0.700	0.563	0.759	0.686	0.857	0.732	0.596	0.811

**Table 10:** Ablation study of attack imperceptibility in terms of hyperparameters used in our method

	SSIM ↑	PSNR ↑	LPIPS ↓
Ours (*a* = 1*, b* = 0)	0.928	31.299	0.118
Ours (*a* = 1*, b* = 0*.*5)	0.932	31.302	0.127
Ours (*a* = 1*, b* = 1)	0.946	31.343	0.108
Ours (*a* = 0*.*5*, b* = 1)	0.950	31.364	0.099
Ours (*a* = 0*, b* = 1)	**0.950**	**31.376**	**0.097**

We further analyzed how parameters *a* and *b* contribute to attack effectiveness and imperceptibility. For example, if *a* = 0, our method selects pixels by focusing only on the imperceptibility. In contrast, if *b* = 0, our method selects pixels by considering only the perturbation ability. Therefore, the worst perturbation performance was achieved for *a* = 1*, b* = 0, whereas the best perceptual attack was obtained for *a* = 0*, b* = 1 in terms of SSIM, PSNR, and LPIPS. Tweaking *a* and *b* can provide a reasonable tradeoff between attack effectiveness and imperceptibility. We leave finding a better tradeoff scheme for our future work.

### Qualitative comparison of perturbed pose and imperceptibility

**Fig. 2:**
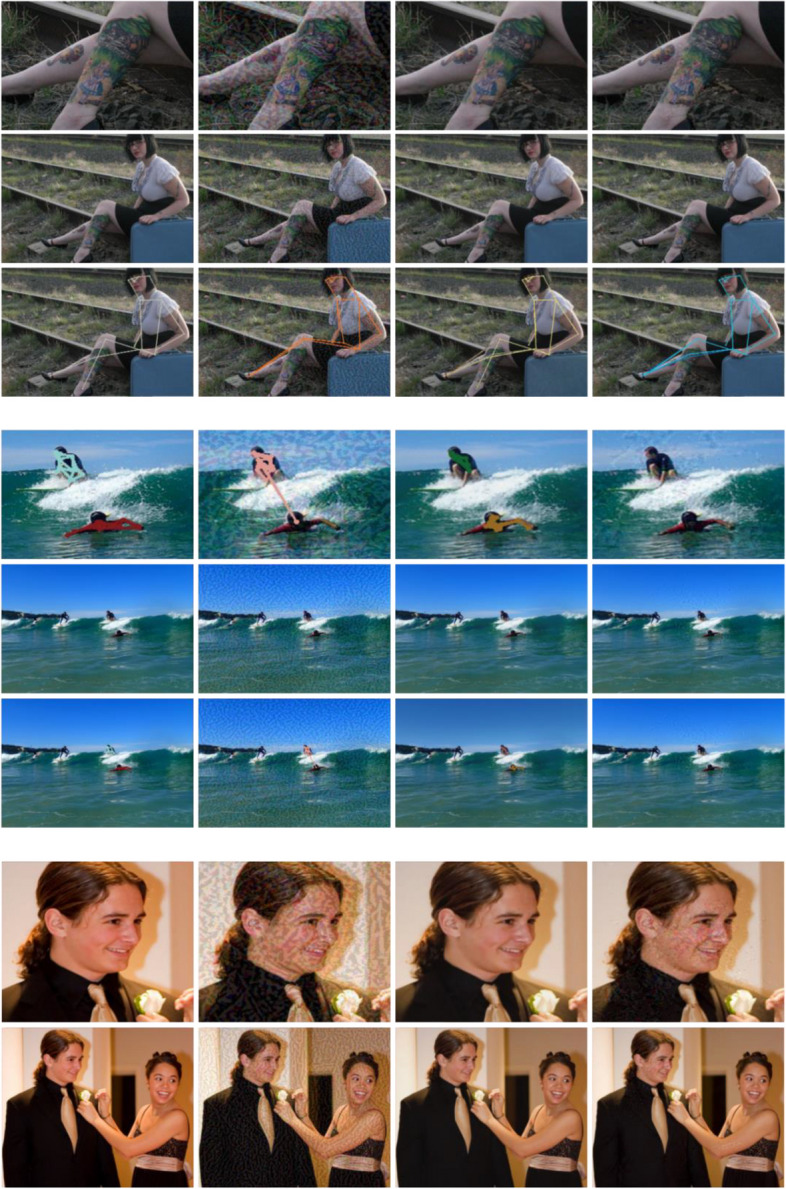
Comparing perturbation crafted by PGD, C&W and ours in various scenarios on HigherHRNet. Adversarial examples of our method are locally perturbed and able to truly maintain imperceptibility. In all cases, PGD and ours can fool HigherHRNet to predict incorrect poses or even to fail in detecting human joints, while C&W cannot always succeed. First column: human poses predicted on clean data. Second column: human poses attacked by PGD. Third column: human poses attacked by C&W. Fourth column: human poses attacked by our method

This section presents a few adversarial samples crafted using the proposed method and baselines. As shown in Fig. 2, we compared the imperceptibility and attack effectiveness of different attack methods. Our adversarial attacks are local and imperceptible to humans and succeed in fooling HPE networks. Although C&W also exhibits good imperceptibility, its attacks are weak and overly smooth for the images. It is obvious that PGD has the strongest attack but the worst imperceptibility and obvious noise. This is because it assumes that the attack strength at every feature dimension is the same, and thus perturbs the entire image. Our local attacks only perturb regions with high variance, which is imperceptible to the human eye. We can see that the proposed locally imperceptible adversarial attack maintains good visual quality for perturbed images without massive noise.

### Discussion

However, the contradiction between attack effectiveness and imperceptibility can be further optimized using more semantic information and body joint features. In future work, we will further reduce the number of critical pixels by incorporating feature maps and the spatial relationships of human-body keypoints.

An HPE attack is essentially a regression-based perturbation method. Therefore, it cannot employ the approach used in classification attacks based on decision boundaries. Instead, it relies solely on a loss function related to keypoint regression to conduct the attack. Our method operates primarily on pixels without considering semantics. In future studies, we aim to incorporate specific action semantics to further enhance the perturbation technique.

## Conclusions

Existing neural networks are vulnerable to adversarial attacks that pose a challenge to the safety of artificial intelligence applications. In this study, we investigated the vulnerabilities of HPE networks and provided an imperceptible adversarial attack against mainstream HPE models. It is generally recognized that attack effectiveness and imperceptibility contradict each other. We optimized this dilemma from both perspectives: theoretical analysis and practical solutions. We formulated the proposed imperceptible attack on HPE networks as a constrained optimization problem using the maximum allowable attack form. This optimization problem can be solved using two alternating suboptimal processes. The first process determines how to refine the perturbation strength, and the second process determines how to select the perturbed pixels. Experimental results demonstrate that the proposed method achieves excellent imperceptibility while maintaining sufficient attack effectiveness.

However, our method does not consider the spatial relationship between feature maps and human-body keypoints. The number of perturbed pixels in the attack is relatively high, and the attack cannot target the physical space.

## Data Availability

Not applicable.

## Abbreviations

C&W: Carlini & Wagner attack
FGSM: Fast gradient sign method
BIM: Basic iterative method
PGD: Projected gradient descent
PSNR: Peak signal-to-noise ratio
SSIM: Structural similarity
LPIPS: Learning perceptual image patch similarity
HPE: Human pose estimation
OKS: Object keypoint similarity
AP: Average precision
AR: Average recall
